# *Chamelea gallina* reproductive biology and Minimum Conservation Reference Size: implications for fishery management in the Adriatic Sea

**DOI:** 10.1186/s40850-021-00096-4

**Published:** 2021-11-25

**Authors:** Giada Bargione, Fortunata Donato, Giulio Barone, Massimo Virgili, Pierluigi Penna, Alessandro Lucchetti

**Affiliations:** 1 National Research Council (CNR), Institute for Biological Resources and Marine Biotechnologies (IRBIM), Largo Fiera della Pesca, 1, 60125 Ancona, Italy; 2 Department of Biological, Geological and Environmental Sciences, University of Bologna, Piazza di Porta San Donato 1, 40126 Bologna, Italy

**Keywords:** *Chamelea gallina*, Reproductive cycle, Sexual maturity, Partial fecundity, Minimum Conservation Reference Size (MCRS), Fishery management

## Abstract

**Background:**

The striped venus clam *Chamelea gallina* is an economically important species in Adriatic Sea fisheries. The use of hydraulic dredging for its catch has a long history in Italy and its management faced several stages of development in the last 40 years. A great effort has been made in the past two decades to move from poorly or weakly managed fisheries to a well-structured co-management system to improve the sustainability of this fishery. However, a prerequisite for appropriate resource management is a sound knowledge of the biology and reproductive strategy of the species.

**Results:**

We investigated three major biological features– the gametogenic cycle, size at sexual maturity and partial fecundity – by microscopic, histological and video analysis techniques. We demonstrated that its breeding season is driven by rises in seawater temperature and chlorophyll-a concentration and that its spawning period lasted from March to September. Size at sexual maturity was reached very early in the life cycle. As regards partial fecundity – the number of mature oocytes potentially released by females with ripe gonads in a single release event – varied in relation to size. Nevertheless, the reduction on the Minimum Conservation Reference Size (MCRS) from 25 to 22 mm (Delegated Regulation (EU) 2020/2237) lead to a 40% reduction in the number of emitted eggs.

**Conclusions:**

We suggest that the ability of Adriatic clam stocks to withstand the strong fishing pressure of the past 40 years and the present one is due to their high reproductive potential and multiple spawning events combined with the effect of management measures (closed areas/seasons, quota, MCRS) and technical constraints on the gear and the sieve on board. Moreover, since the reduced MCRS for Venus shells is still larger than the size at maturity, it will probably not be detrimental to the reproductive capacity of the stock.

**Supplementary Information:**

The online version contains supplementary material available at 10.1186/s40850-021-00096-4.

## Background

The striped venus clam (*Chamelea gallina* Linnaeus, 1758) is an economically important species in the Mediterranean Sea, where it thrives at depths of 2–12 m [1, 2] in the coastal biocenosis of well-sorted fine sands described by Pérès and Picard [3], although it can even reach greater depths up to 20 m [4, 5]. In Italy, the fleet targeting *C. gallina* consists of 635 active hydraulic dredges, 601 of which are concentrated along the Adriatic coasts [6]. Hydraulic dredges harvesting *C. gallina* operate in a narrow strip between 0.3 and 2 nautical miles (NM) off the coast (depth range 3–15 m), and along 1400 km of the 8000 km of the Italian coastline. In the early 1970’s, the transition from hand-operated to hydraulic dredges resulted in an immediate and steep yield increase of up to 80,000–100,000 tons/year that was followed by a progressive decline due to overexploitation and poor management [7]. In the past two decades, considerable effort has been made to move to a well-structured co-management system, to improve the sustainability of this fishery [8]. In recent years, annual production has been around 19,000 tons (~ €51.4 million), accounting for 11% of fishery production in Italy in weight and for 6% in revenues [6]. However, the declining landings do not accurately reflect the status of the resource at sea, which is influenced by factors such as natural population fluctuations; the variable catch quota, which is set on the basis of market demand; and the wide range of restrictions that have been adopted over time to promote the sustainability and responsiveness of the fishery. The *C. gallina* fishery is managed through technical measures that:

- set dredge dimensions (maximum width, 3 m; maximum weight, 600 kg; Ministerial Decree 22/12/2000 [9]);
- regulate the fishing effort through closed areas (dredging is banned within 0.3 NM of the coast; Regulation (EC) 1967/2006 [10]);
- ban fishing activities in some periods (dredging is forbidden for 2 months between April and October [6]);
- limit the catch of juveniles through constraints on the technical features of the dredge and the mechanical sieve on board (Ministerial Decree 22/12/2000) [9];
- establish the Minimum Conservation Reference Size (MCRS)*,* which is currently 22 mm (Delegated Regulation (EC) 2016/2376, Regulation (EC) 2020/3, and Delegated Regulation (EC) 2020/2237 [11–13]) by way of derogation from the previous 25 mm (Annex III to Regulation (EC) 1967/2006 [10]).

A prerequisite for appropriate resource management is a sound knowledge of its biology and reproductive strategy. The reproductive cycle of *C. gallina* in the Adriatic has been reported to span from March to September [14–16]. However, there is disagreement on its size at sexual maturity (TL_50_), which has been described to range from 11 to 18 mm [4, 17, 18]. A thorough knowledge of this parameter is crucial to evaluate the spawning fraction and fecundity of the population that has not been harvested, which contributes to its reproductive output [19].

*Chamelea gallina* reproduction has been the subject of several qualitative studies in the Adriatic Sea and elsewhere [20–24]. On the other hand, there is only one quantitative study investigating the potential number of emitted eggs per females in a single spawning event in relation to shell size [19]. The disproportion is due to the diffusion of gonad tissue in the visceral mass, which hampers the study of reproductive output and investment in all bivalves [25] except Pectinidae, whose gonad is a discrete organ. Quantitative reproduction data, like gonad biomass and fecundity, are critical to understand the life history of marine bivalves and to manage them successfully, i.e. to define the MCRS [26–28].

Various semi-quantitative and quantitative methods have been applied to estimate bivalve fecundity also in relation to their reproductive strategy and ovary structure, even though quantitative investigations are still much fewer than qualitative studies [29]. For example, the reproductive investment has been assessed in live specimens by inducing spawning through thermal shock or chemical injection, to count the number of eggs released [30–32] or by weighing them before and after spawning [33]. Dead specimens can be analysed indirectly by strip spawning [34, 35], volumetric reconstruction [36–38] and histological [39] and immunological methods [40]. However, all of them underestimate the reproductive output, since incomplete spawning is not infrequent and spawning events of different intensity may occur several times during the reproductive season [41]. This is the case of *C. gallina*, a multiple partial spawner with intraindividual asynchronous ovary development [42]. Bivalve fecundity is closely related to size and age [7, 38], although it can also be influenced by phylogeny and environmental conditions [25, 43–45].

Altogether, the information on the reproductive biology of *C. gallina* in the Adriatic Sea is dated and limited, which has the potential to undermine stock management and conservation efforts. The aim of this study is to provide new and updated information on the reproductive cycle of *C. gallina*: *i)* by investigating the gametogenic cycle using histological techniques and evaluating its relationships with temperature and chlorophyll-a (Chl-a); *ii)* by estimating TL_50_ by microscopic observation; and *iii)* by assessing partial fecundity (PF) by means of histological and image analysis approaches. Since in Italian territorial waters the MCRS has temporarily been reduced from 25 mm to 22 mm total length (TL) we also describe how the reduction affects clam fecundity. The study findings provide insights for fishery management, such as the MCRS and the time of the year when fishing should be closed.

## Results

### Environmental parameters

**Fig. 1:**
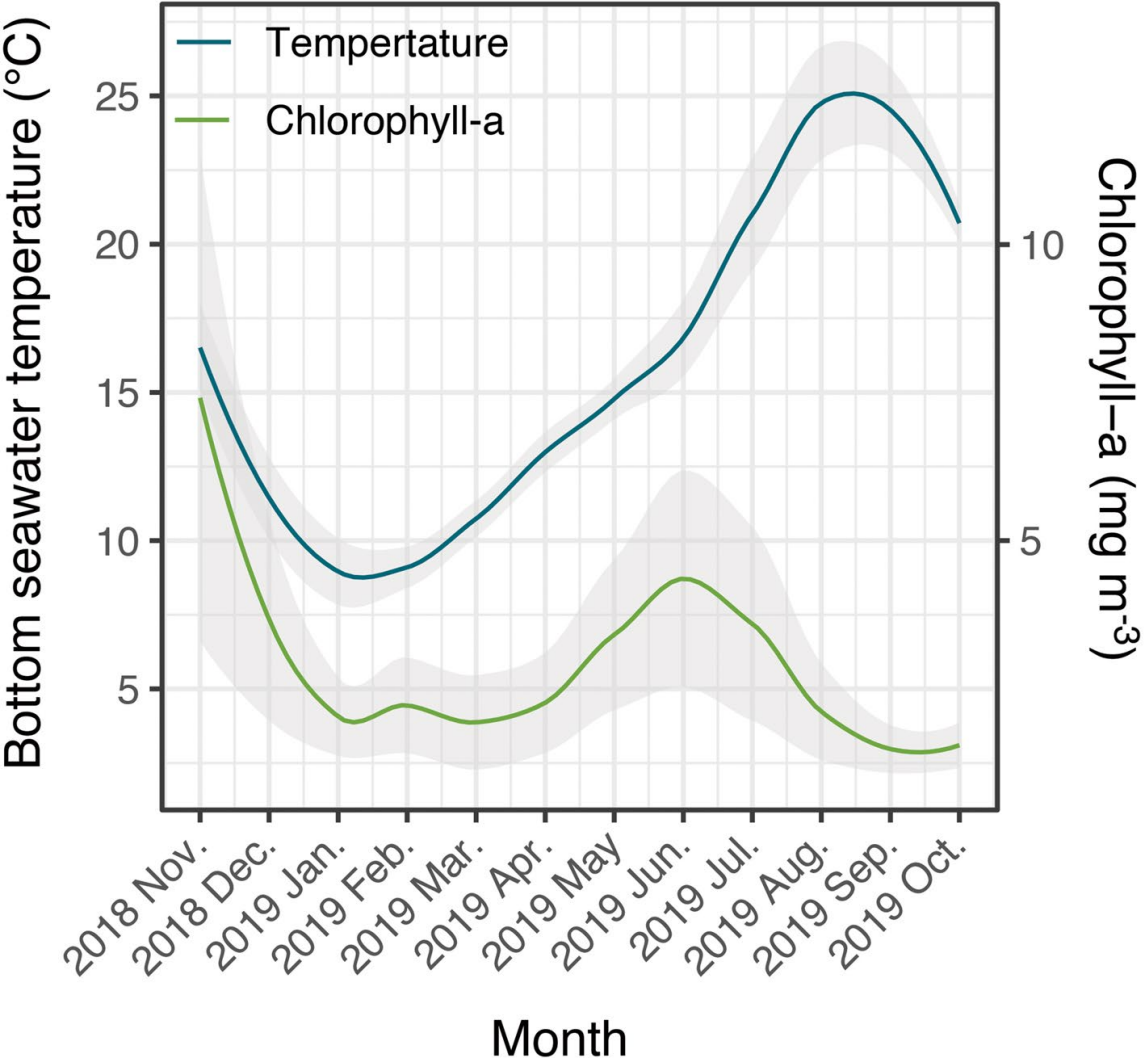
Mean monthly bottom seawater temperature and chlorophyll-a concentrations recorded between November 2018 and October 2019. Grey area: standard deviation

The mean monthly BST and Chl-a values exhibited a seasonal trend (Fig. 1). BST was 16.6 ± 1.4 °C in November, dropping to 10.7 ± 0.5 °C until March with a minimum at 8.8 ± 1.1 °C in January. It gradually rose from January, and in June it approached the values recorded in November (16.4 ± 1.0 °C). From July to October, BST ranged from 20.6 ± 2.2 to 25.8 ± 1.5 °C, peaking in August. Chl-a fell steeply from 7.8 ± 4.3 mg/m^3^ in November to ~ 2.2 ± 0.8 mg/m^3^ in December–April, it increased in May–July with a peak in June (4.6 ± 1.9 mg/m^3^) and fell again from August to October (1.6 ± 0.4 mg/m^3^).

### Histology

**Fig. 2:**
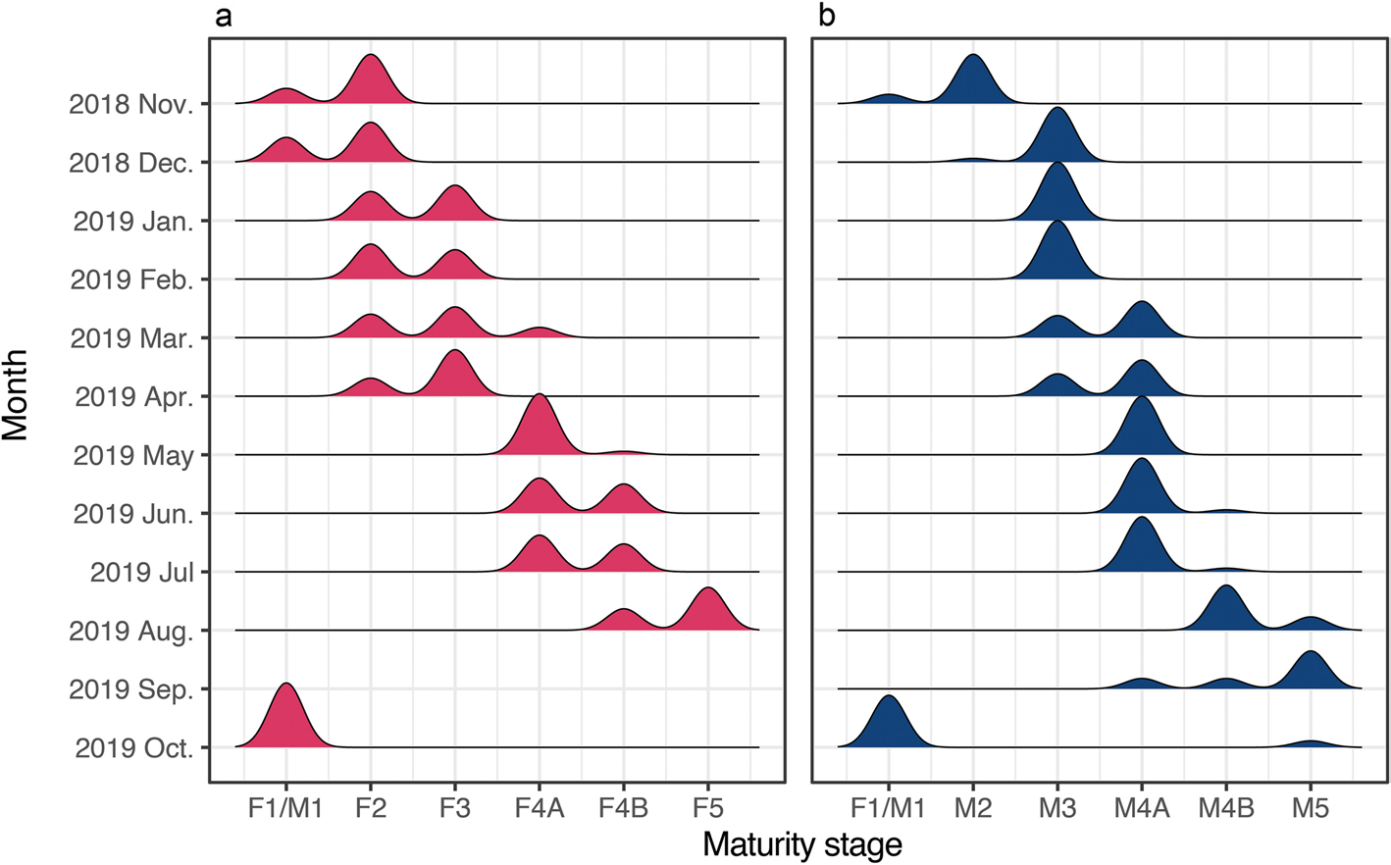
Ridge plots showing the progress of maturity stages over the months in **a** females and **b** males. The curves illustrate the percentage of individuals in each maturity stage. The sum of the areas defined by the curves corresponds to the total monthly observations

**Fig. 3:**
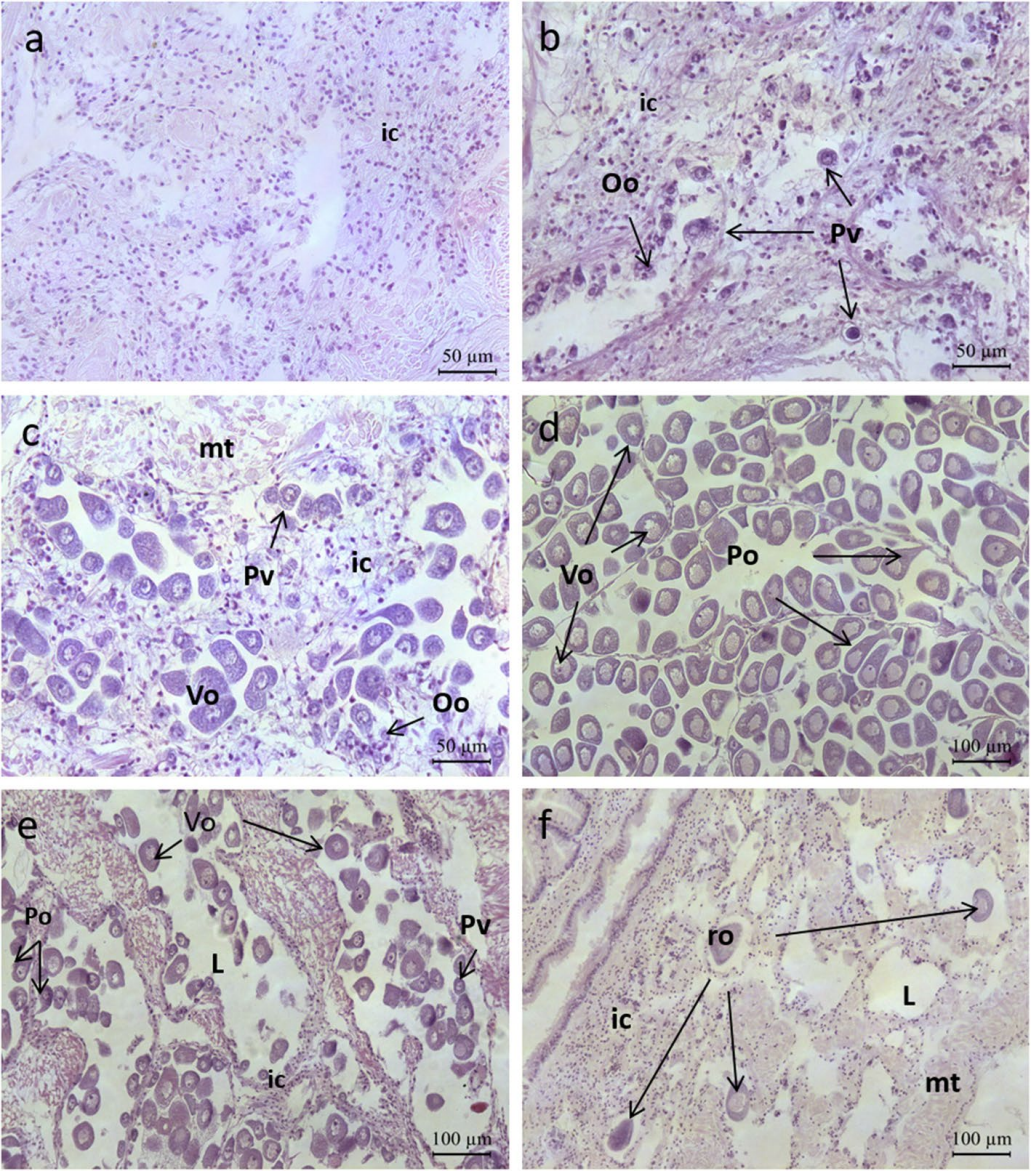
Histomorphological maturity stages in *C. gallina* females: **a** inactive stage, M1/F1; **b** early active stage, F2; **c** late active stage, F3; **d** ripe stage, F4A; **e** partial emission stage, F4B; **f** regressing stage, F5. Abbreviations: ic: immature cells; Oo: oogonia; Pv: previtellogenic oocyte; Po: pedunculated oocyte; Vo: vitellogenic oocyte; L: lumen; ro: residual oocyte, mt: muscle tissue

**Fig. 4:**
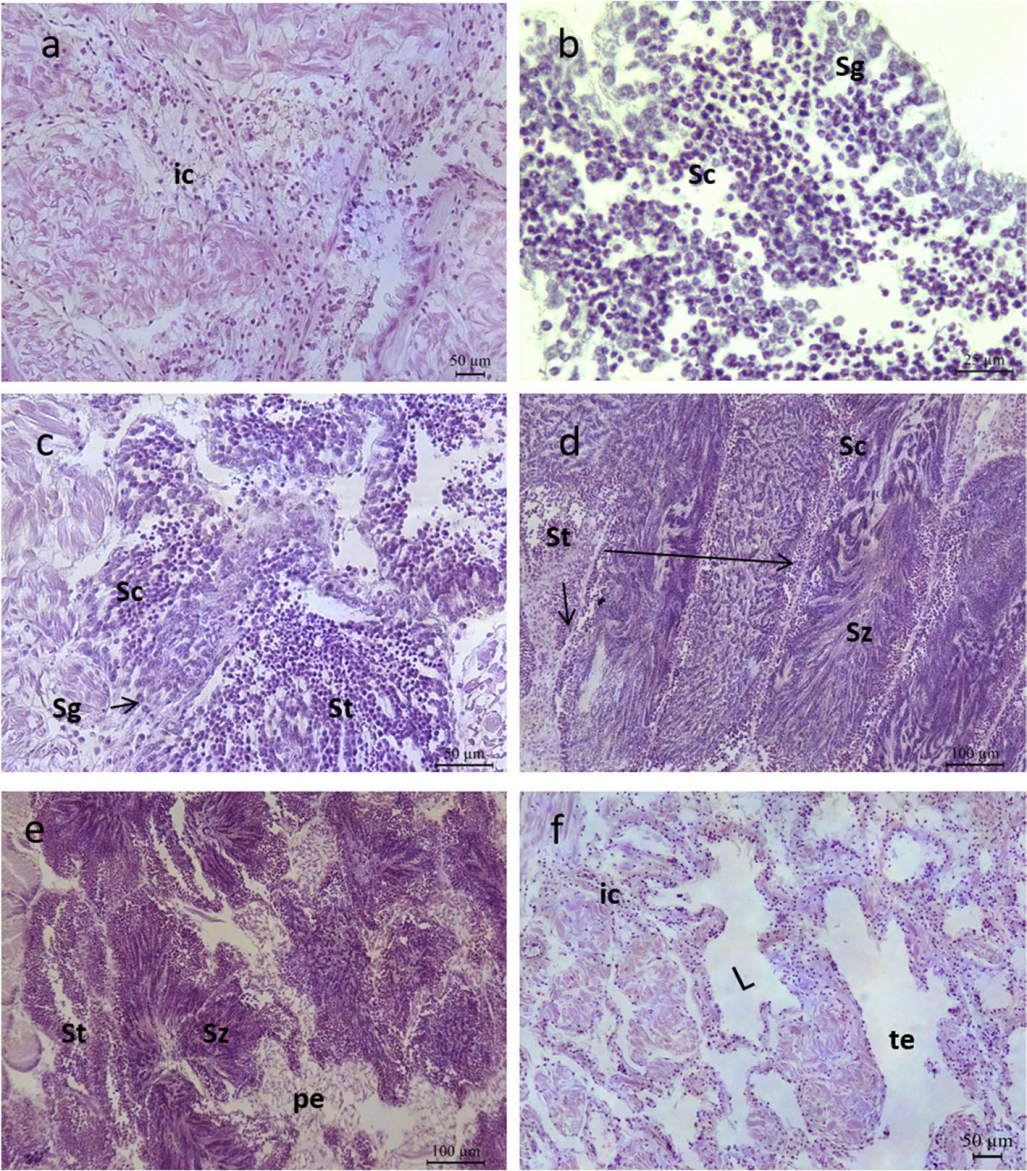
Histomorphological maturity stages in *C. gallina* males: **a** inactive stage, M1/F1; **b** early active stage, M2; **c** late active stage, M3; **d** ripe stage, M4A; **e** partial emission stage, M4B; **f** regressing stage, M5. Abbreviations. ic: immature cells; Sg: spermatogonia, Sc: spermatocytes; St: spermatids; Sz: spermatozoa; pe: partial emission; te: total emission; L: lumen

Altogether 213 females, 205 males and 64 indeterminate individuals were subjected to histological analysis. The progress of maturity stages over the months is reported in Fig. 2. During the 12-month sampling, the 64 indeterminate individuals were recorded only in November, December and October. At this stage sexes were not distinguishable, therefore the inactive stage is hereafter reported as F1/M1. The inactive stage was characterized by abundant connective tissue occupying the whole visceral mass without follicles/acini or gametes; only indeterminate cells were present and the sexes were indistinguishable (Figs. 3 and 4a).

In November gametogenesis had already resumed, as 56.5 and 76.7% of females and males were respectively in stage F2 and M2 (early gametogenesis). The gonads of F2 females showed fully developed follicles and oogonia (diameter, 5–6 μm) together with some small previtellogenic oocytes in early stage of development (diameter, 15–20 μm) around the follicle wall; only vesicular cells were seen in the lumen (Fig. 3b). This stage was predominant in November and December (~ 60%), whereas in January–March an almost equal proportion (~ 50%) of females were in stages F2 and F3. In April, 72% of females were in stage F3 (late active stage) and 28% were still in stage F2. Stage F3 oocytes were considerably larger and most were in the previtellogenic and pedunculated stages. Pedunculated oocytes protruded into the lumen of the follicle through their stalk, whereas a small number of vitellogenic oocytes were seen free in the lumen (Fig. 3c). In May, 95% of females had ripe gonads (F4A stage) with pedunculated and vitellogenic oocytes filling the lumen (Fig. 3d). In March, a small fraction of females (14%) were already in the ripe stage. Evidence of partial release (F4B, partially spawned stage) was detected since May (5%); this stage became more represented in June and July, when females with full or partially empty gonads were in almost equal proportion (~ 50%). The partially empty follicles indicated that a first release event had already occurred. In F4B females new and residual pedunculated and vitellogenic oocytes were detected in the lumen together with oocytes in an earlier stage of development attached to the wall, indicating the resumption of gametogenesis (Fig. 3e). In August, 67% of females had regressing gonads (stage F5) with residual oocytes in the collapsed lumen and connective tissue and indeterminate cells surrounding the gonad area (Fig. 3f). In September, we collected a single female (stage F5). In October, the gonads had completely regressed and females could no longer be identified, since all individuals were in the inactive stage (M1/F1).

In November, males were in the early gametogenesis stage (M2) with fully formed acini, spermatogonia surrounding the walls and vesicular cells filling the lumen (Fig. 4b). Between December and February, males were almost exclusively (94–100%) in late active gametogenesis (stage M3), with germ cells of decreasing size – spermatocytes and spermatids – arranged centripetally and projecting into the lumen (Fig. 4c). From March to July, most males (67–100%) had ripe gonads (stage M4A), with the lumen of the acini filled with spermatids and spermatozoa (Fig. 4d). Partial release events (stage M4B) spanned from June to September, peaking in August (84.5%). In M4B males, new spermatids and spermatozoa occupied a portion of the partially emptied acini, although cells in earlier development stages were also detected along the acinar walls (Fig. 4e). Gonad regression (stage M5) began in August, the majority of males in this stage being observed in September (73.9%); the acini were collapsed, with connective tissue and indeterminate cells beginning to surround the gonad area; residual spermatozoa were present except where a total release event had occurred (Fig. 4f). In October, a residual of 0.7% of males were still in regression, whereas all the other individuals (97.3%) had inactive gonads.

### Size at sexual maturity

**Fig. 5:**
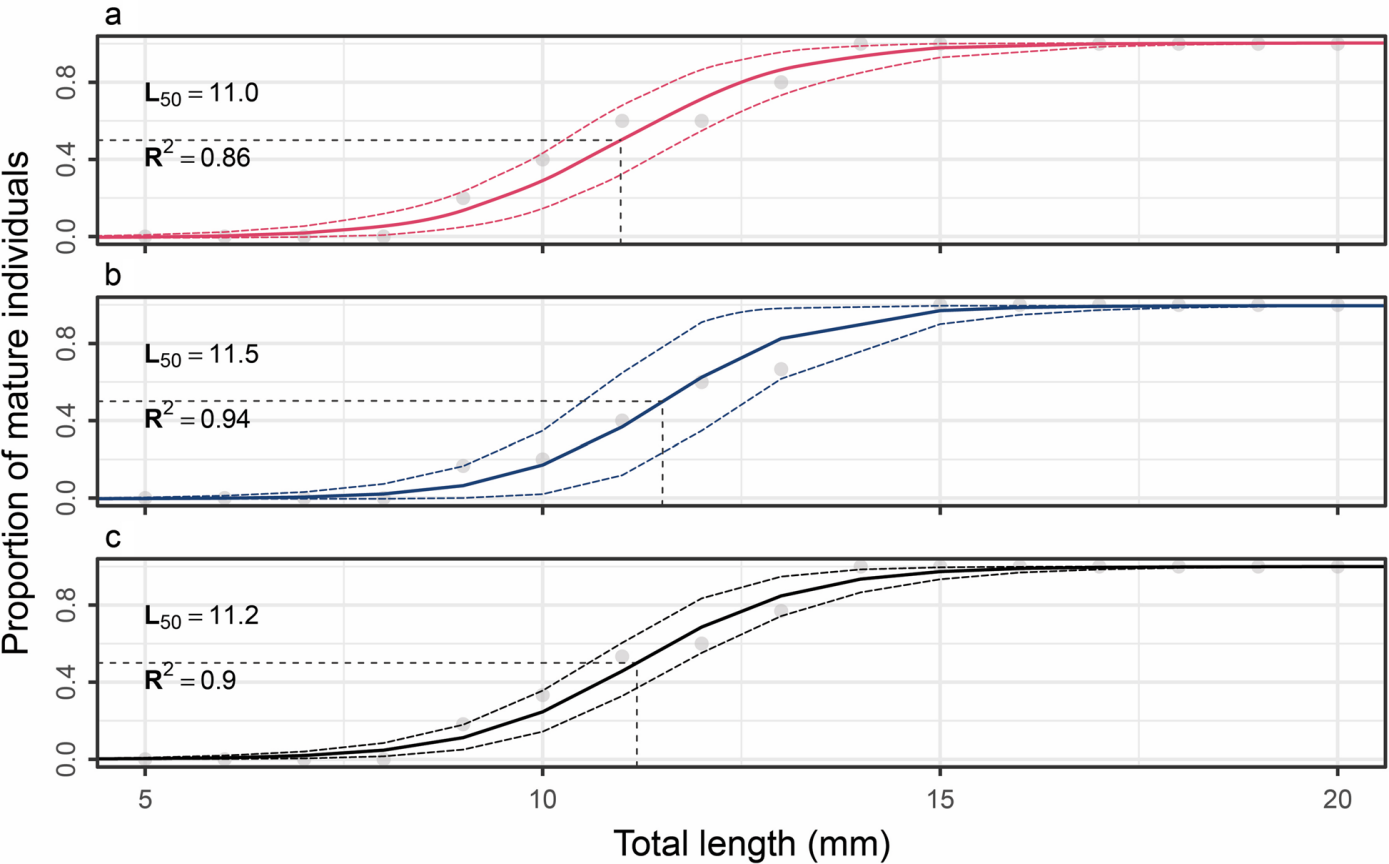
Size at sexual maturity assessed in *C. gallina* **a** females, **b** males and **c** pooled sexes

A total of 504 additional individuals (227 females, 243 males and 34 indeterminate), collected during the ad hoc sampling carried out in the middle of the reproductive season, were analyzed to assess TL_50_ in both sexes. The smallest females and males with well-developed gametes measured 9.6 mm and 9.9 mm TL, respectively; TL_50_ was ~ 11.0 for females and 11.5 mm for males, whereas the TL_50_ of the entire sample was ~ 11.2 mm (Fig. 5). Above 15 mm TL, all males and females were sexually mature.

### Partial fecundity

The gonad volume ranged from 25.2 to 280 mm^3^ and was significantly and positively related to size (G_v_ = 17.2 × TL – 304.5; adj. R^2^ = 0.97; F_1,24_ = 772.5; *p* < 0.001). The percent G_v_ occupation by all types of oocytes was significantly different between maturity stages (ANOVA, F_1,24_ = 64.4; *p* < 0.001) and was 39.6 and 21.8% in stage 4A and stage 4B, respectively. Two-way ANOVA, testing for the effect of Maturity stage and Oocyte development stage on the percent G_v_ occupation, highlighted significant differences between the two parameters and their interaction. Although the interaction was statistically significant (Table 1), the G_v_ occupied by mature and immature oocytes (19.1 and 20.5%, respectively) in 4A females was not significantly different, whereas in 4B females immature oocytes occupied almost twice the volume compared with mature oocytes (14.0 and 7.8%, respectively; Table 1).

**Table 1:** Results of two-way Analysis of Variance (ANOVA) and the Tukey HSD test for the effects of Maturity stage (4A and 4B), Oocyte development stage (Mature / Immature), and the interactions of the two terms

	**Df**	**Sum Sq**	**F value**	**Pr(>F)**
**ANOVA**				
Maturity stage	1	976.2	76.342	< 0.001^*^
Oocyte stage	1	136	10.637	< 0.01^*^
Maturity stage * Oocyte stage	1	72.2	5.645	< 0.05^*^
Residuals	48	613.8		
	**diff**	**lwr**	**upr**	**p adj**
**Tukey HSD test**				
4B - 4A	−8.906	−10.955	−6.857	< 0.001^*^
Mature – Immature	−3.235	−5.230	−1.241	< 0.01^*^
4B Immature - 4A Immature	−6.484	−10.321	−2.648	< 0.001^*^
4A Mature - 4A Immature	−1.372	−4.736	1.993	0.7
4B Mature - 4A Immature	−12.699	−16.536	−8.863	< 0.001^*^
4A Mature- 4B Immature	5.112	1.276	8.949	< 0.01^*^
4B Mature - 4B Immature	−6.215	−10.471	− 1.959	< 0.01^*^
4B Mature- 4A Mature	−11.328	−15.164	−7.491	< 0.001^*^

^*^indicate significant effects for *P* values < 0.05

**Fig. 6:**
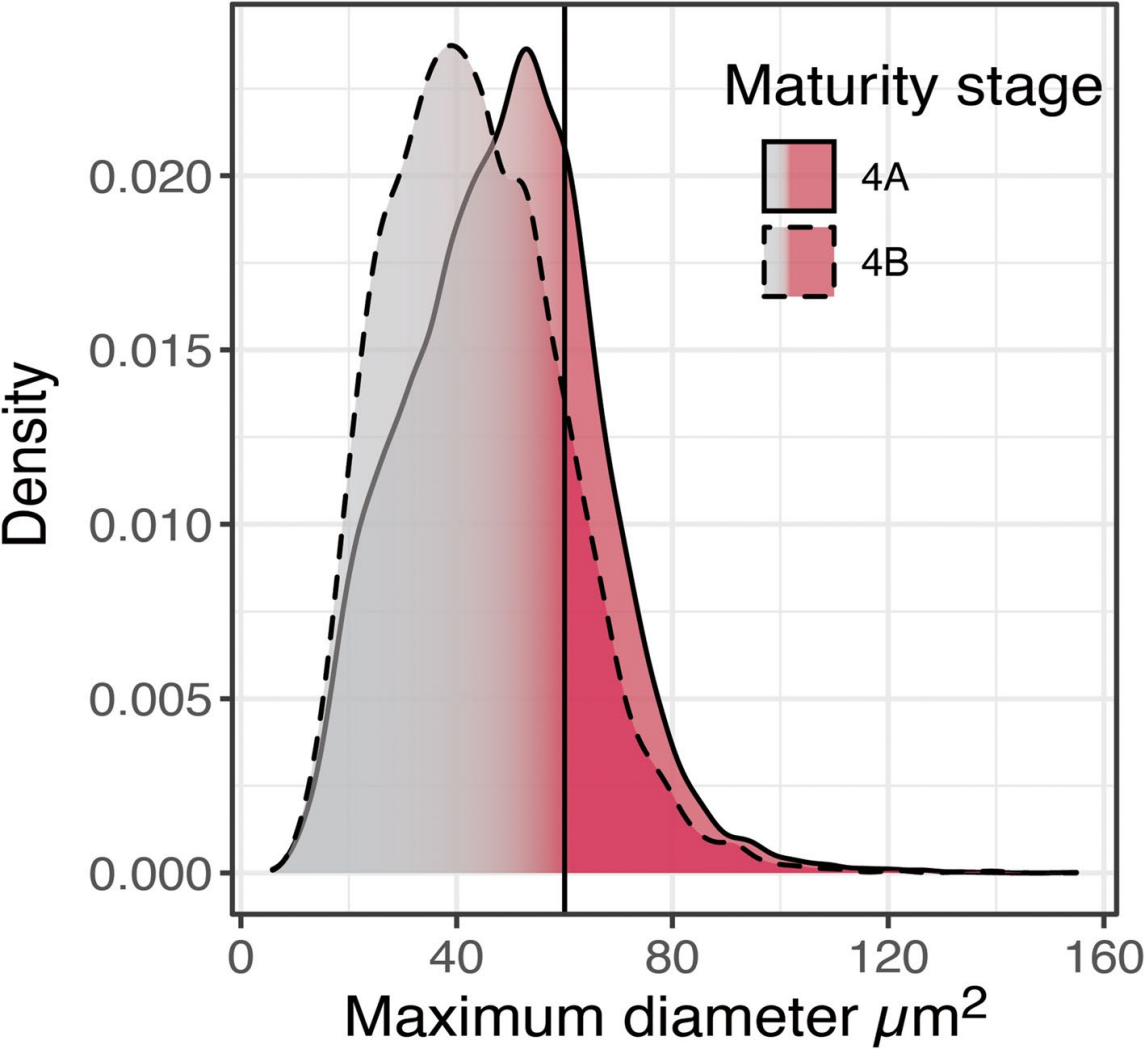
Size frequency distribution of maximum oocyte diameter in 4A and 4B females. The vertical line divides oocytes into those larger and smaller than 60 μm in maximum diameter

The size frequency distribution of oocyte d_max_ between the two maturity stages showed that the mode was 53 mm in stage 4A and 41 mm in stage 4B (Fig. 6). In mature oocytes, the largest d_max_ values were 154.89 μm and 139.21 μm in stages 4A and 4B, respectively, and the smallest d_max_ values were 5.85 μm and 9.54 μm, respectively. The mean diameter of mature and immature oocytes was respectively 70.3 μm, 41.5 μm in stage 4A and 70.1 μm and 38.6 μm in stage 4B.

**Fig. 7:**
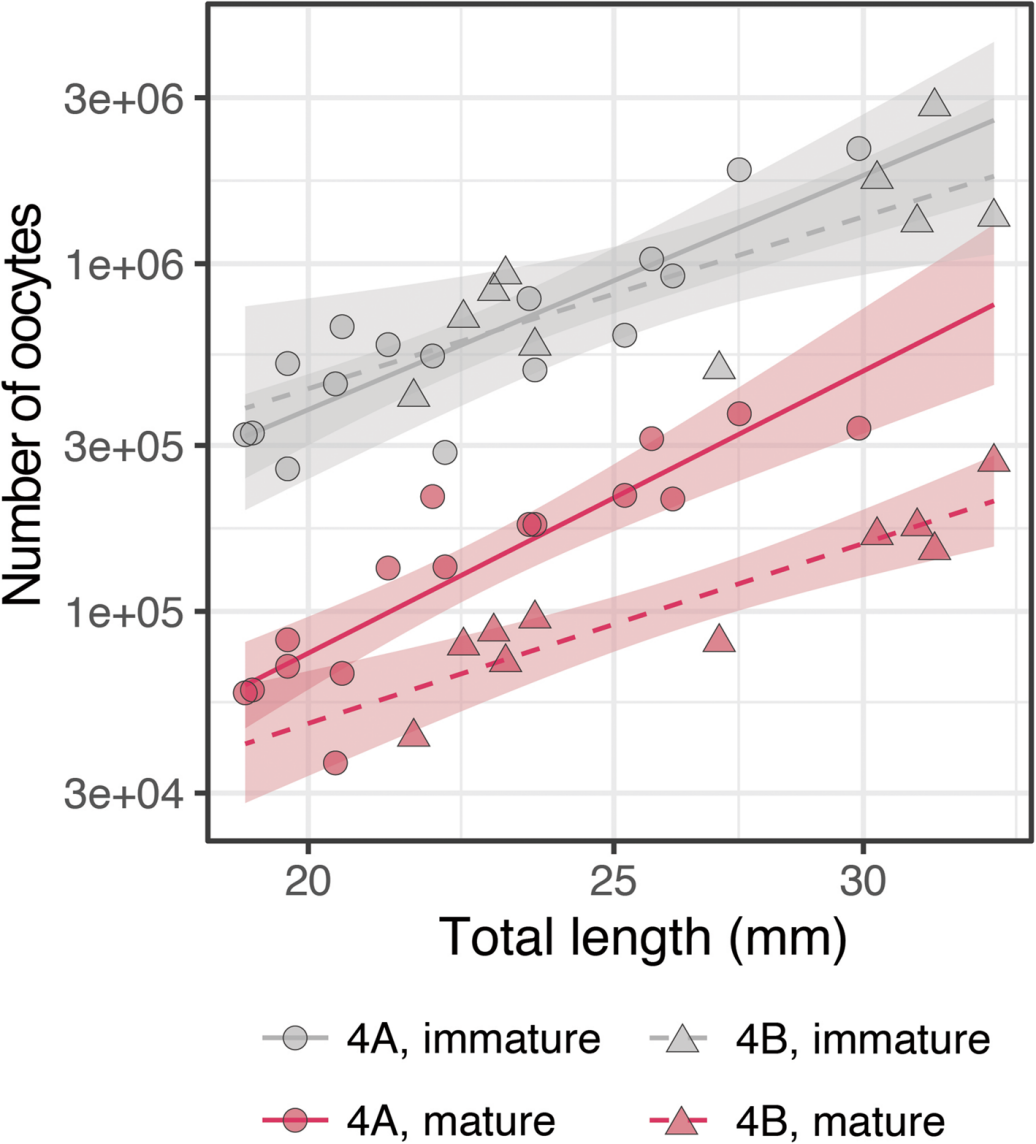
Scatterplots illustrating the relationship between oocyte number and total length. The log-scale emphasizes the differences between and within maturity stages (4A and 4B) in terms of oocyte maturity (mature / immature)

There was a strong positive and significant linear relationship between the number of any type of oocytes and TL, irrespective of maturity stage (Fig. 7). Two-way ANCOVA indicated that, while controlling for TL, there was a significant difference in the total number of oocytes between 4A and 4B females (ANCOVA, F_1,47_ = 121.638; *p* < 0.001). Similarly, there was a significant two-way interaction between Maturity stages and Oocyte development stages in the number of oocytes while controlling for TL (ANCOVA, F_2,47_ = 186.131; *p* < 0.001). A simple main effects test for Maturity stage and Oocyte development stage demonstrated that mature oocytes were more numerous in 4A than 4B females (F_1,23_ = 30.8; *p* < 0.001), whereas the difference between 4A and 4B females in terms of number of immature oocytes was not significant (F_1,23_ = 0.185; *p* < 0.671).

In 4A mature females size ranged from 19.2 to 29.9 mm TL with a PF from 3.6 × 10^4^ to 3.7 × 10^5^ oocytes/female depending on size (average, 1.6 × 10^5^ oocytes/female). The linear regression analysis (PF = 3.01 × 10^4^ TL − 5.21 × 10^5^; adj. R^2^ = 0.85; F_1,14_ = 83.8; *p* < 0.001) suggested that 4A females can release ~ 1.4 × 10^5^ (95% confidence interval, CI ± 2.3 × 10^4^) oocytes/female at size 22 mm TL (present MCRS) and 2.3 × 10^5^ (95% CI ± 2.7 × 10^4^) oocytes/female at size 25 mm TL (ex-MCRS).

## Discussion

### Reproductive biology

This study describes the year-round reproductive cycle of the commercially valuable species, *C. gallina*, in the western Adriatic Sea. The gonad development of *C. gallina* exhibits a cyclical annual pattern influenced by BST and Chl-a. In November, when we began sampling, gametogenic activity was already detectable and an important fraction of females and males were in the early active stage (F2/M2). In November, the high Chl-a concentration and the high BST, which was similar to the one recorded in June (~ 16 °C), when the clams were spawning, may have acted as a trigger. Indeed, temperature abnormalities (> 14–18 °C) have been suggested account for the advanced stage of maturity and reproduction seen in clams in autumn and winter [15, 46, 47]. In temperate climates the most common bivalve gametogenesis pattern is initiated by the seawater temperature reaching a certain threshold [48].

High energy stores in late autumn – related to the high Chl-a concentration detected in November – combined with high BST values, probably drive gonad development to the next stage (F3/M3). In December, Chl-a and BST both dropped; in January and February – the two coldest months, with BST under 10 °C – gonad maturation stopped. The percentage of females and males in the different stages of maturity remained almost unchanged. Our observations agree with studies indicating that clam growth [49] and gonad development [50] slow down when BST is less than 10 °C. In March, when BST exceeded 10 °C and Chl-a began to increase, gametogenic activity resumed and ripe gonads were first detected, especially in males. Most spawning events, highlighted by evidence of partial release and gonad recovery, occurred from May to August as both BST and Chl-a rose.

In August, when BST peaked, some clams began to show gonad regression (F5/M5). This stage was predominant in September and was followed by the inactive stage (F1/M1) in October. Similarly, a study of *Ensis arcuatus* in north-western Spain highlighted that the last spawning event before gonad regression was associated with an increase in surface temperature [51]. Several studies have demonstrated that water temperature and food availability significantly influence the reproductive cycle of *C. gallina* [19, 22, 24] and other bivalves [51–53]. This is especially true in temperate regions, where increasing temperature and food supply accelerate gonad development in numerous clam species [54–56]. Indeed, the striped venus clam shows an opportunistic reproductive strategy, since gonad development and sexual maturation are closely associated with nutrient accumulation, i.e. food availability [29, 57]. Whereas we detected two Chl-a peaks, one in early autumn and the other in late-spring/early-summer, in other temperate areas Chl-a peaks in late autumn [24] or in late summer and winter [19], despite similar seasonal seawater temperature patterns. However, bivalve reproductive activity is controlled not only by environmental factors, but also by their interaction with endogenous processes [53, 57, 58].

In recent years, the reproductive cycle of *C. gallina* has widely been investigated, especially along the Spanish, Portuguese and Turkish coasts, whereas the majority of studies in the Adriatic are fairly dated (Additional file 1). An extended spawning period has been described by most studies in all areas [14, 23, 24, 59], although a shorter period has also been reported [22, 47, 50]. In the Adriatic Sea, the reproductive cycle of *C. gallina* commonly spans from March to September, with some additional reproductive events in early autumn, whereas studies conducted at different temperate latitudes have described reproductive events only from late spring to late summer (Additional file 1). The reproductive plasticity of *C. gallina* can be explained by changes in local environmental and trophic conditions over time and by geographical location [46, 57]. In temperate areas eggs are released in favourable conditions for the development of planktotrophic larvae, when phytoplankton and Chl-a concentrations are abundant and when the water temperature ranges from 18 to 27 °C [15].

In the present study, evidence of partial gamete release and developing gametes in the same acinus/follicle in 4B individuals, heralded further spawning events as long as environmental conditions would be favourable for reproduction within the same reproductive season, otherwise gametes are reabsorbed at the end of it. These findings confirm that the striped venus clam is a multiple partial spawner [19–21, 42], even though single spawning events have been described by other authors [17, 24, 60]. In our study, all specimens were gonochoric, albeit cases of hermaphroditism have been reported [7, 21]. Gonad development was synchronous in females and males, as reported in several studies [24, 50, 59], a strategy that maximizes reproductive success. Another well-established feature of C. gallina is interindividual asynchrony, whereby specimens in different maturity stages coexist in the same period (Additional file 1). In contrast, intraindividual asynchrony – where different maturity stages coexist in the same individual – has rarely been described before the present study [19–21].

Both sexes of the striped venus clam reach sexual maturity at about 11.2 mm TL, in the first year of life; indeed, the first year specimens grow to about 15 mm TL [61]. Clams longer than 15 mm TL were all found sexually mature. Our findings are consistent with previous studies reporting a similar or even smaller size at sexual maturity [18, 46, 59, 62–64]. Yet, a TL_50_ of 9 to 18 mm is commonly described in the Adriatic Sea and elsewhere (Additional file 1). Such different values, reported even in the same area, may be attributed to the intrinsic reproductive variability of the species in relation to local environmental conditions such as seawater temperature, food availability and to anthropogenic, genetic and physiological factors [57], as well as to the different methods adopted to asses maturity.

Although the estimation of potential annual fecundity is critical to understand bivalve production and population dynamics, it is little explored [65]. *Chamelea gallina* is characterized by indeterminate fecundity, or better by a potential annual fecundity that is not known before the onset of spawning, since unyolked oocytes continue to mature and be spawned throughout the reproductive season [66]. Even though we were able to estimate the PF related to a single egg release event, in multiple partial spawning bivalves the number of spawning events occurring in the same reproductive season and the intensity of each reproductive peak are unknown [41], and are different in different years [67]. Only another study by Delgado et al. [19], conducted in the Gulf of Càdiz (south-western Spain), has assessed the fecundity of *C. gallina.* The results of the two investigations are quite similar; in particular, Delgado and co-workers analysed females in a size interval (20–30 mm TL) similar to ours, they found similar estimates of gonad volume (range, 37.25–205.95 mm^3^) and reported that the percent G_v_ occupied by all types of oocytes and by mature oocytes was respectively 37.71 and 18.38% in 4A females and 31.30 and 14.23% in 4B females. Nevertheless, their estimated PF (range, 7.6 × 10^4^–7.9 × 10^5^ oocytes/female) is higher than ours, despite a similar order of magnitude of oocyte number in relation to TL. The difference may lie in the method used to calculate PF: we only considered oocytes sectioned through the nucleus, which involves that the actual number of oocytes in the gonad may have been underestimated, whereas Delgado et al. [19] did not report it. Before egg release, the oocytes can reach a diameter of 110–120 μm [18], which is comparable with the d_max_ values we found in 4A and 4B mature oocytes. We found that fecundity is related to size, as noted by other authors [38, 44, 68], since in younger individuals growth is fast and the investment in reproduction limited, whereas in older bivalves energy is switched from growth to reproduction [44, 45].

A variety of studies have tried to estimate fecundity in various bivalve species, despite the problem of gonad tissue diffusion in the visceral mass. For example, in *Spondilus calcifer* the mean number of spawned oocytes per female has been estimated at 48.9 million [68], whereas the number of eggs per female has been put at 4.15 million in *Ruditapes philippinarum* [41] and at 1.65 million in *Anadara antiquata* [38]. The order of magnitude of the mean number of spawned eggs per female, reported in these studies, is up to two times higher than the one we calculated. However, egg number strongly depends on the species, its size range and the estimation method.

The reproductive strategy of *C. gallina* results in high fecundity. As near-sessile organisms, their lifecycle is strongly affected by environmental factors [36]. To ensure reproductive success, large amounts of gametes are released in the water column and, after fertilization, develop into planktotrophic larvae [15, 69]. However, as demonstrated by Beninger et al. [65] in *Cerastoderma edule* using Neutral Red vital staining, not all the oocytes released during a spawning event are viable, as dead/non-viable oocytes accounted for 34–85%. Moreover, oocytes age after spawning and 4–8 h after their release they can no longer be fertilized [70]: this involves that synchronization of gamete release in the environment is crucial for the reproductive success of the species [7]. Egg number is further reduced by predation by filter-feeding organisms in the water column. In addition, early offspring mortality is also substantial, due to oceanographic and ecological factors (e.g. food availability, current transport to unsuitable habitats, predation [71];) as well as to biological (e.g. reproductive strategy of the species, larval duration and larval behaviour [72];) and genetic factors [43].

### Management implications

Italian clam fishery is the sole fishery where the number of vessels and operators has not declined in the past four decades [6]. The biological and management factors that allowed the clam fishing stocks to withstand the high fishing effort include:

i) the high reproductive potential (clams of 22 mm TL produce 1.4 × 10^5^ oocytes/female, a fairly high fecundity whose order of magnitude is shared with 25 mm clams) and the multiple spawning events occurring within the same reproductive season;
ii) the early maturation, since all clams > 15 mm TL are sexually mature within the first year of life;
iii) the closure of the area within 0.3 NM of the coast (Regulation (EC) 1967/2006 [10]) to dredging activity; this measure has halved the area previously suitable for clam harvesting and provides a large area (581.7 km^2^) where a huge amount of breeders contribute to the reproductive output of the population;
iv) the daily quota (reduced to 400 kg/vessel from the previous 600 kg/vessel; Delegated Regulation (EC) 2016/2376 [12]) has strongly reduced the fishing effort, because the boats take less time to achieve the predetermined quota;
v) the two-months fishing closure adopted in summer during the peak of reproduction;
vi) the technical measures set for the fishing gear (for both the dredge and the sieve on board) reduce the catch of juveniles and the fraction below 22 mm TL almost to zero [73];
vii) the setting of restocking areas, entered into force in 2017, where fishing is banned and where fishermen are required to discard undersized specimens harvested elsewhere (Delegated Regulation (EC) 2016/2376 [12]);
viii) the high survival rate of *C. gallina* (higher than 95% [74]); the specimens returned to the sea can grow and contribute to the spawning fraction of the population;
ix) the seeding and fishing area rotation applied by Management Consortia, the bodies responsible for fishery management, make the exploitation more sustainable and responsive.

The Scientific, Technical and Economic Committee for Fisheries (STECF), in the Joint Recommendation 20–01, reported that since the first implementation of the new MCRS in 2017 (Commission Delegated Regulation (EC) 2016/2376 [12]) an increase of abundance of > 22 mm individuals has been observed in the stock in certain areas of the Adriatic Sea. STECF also noted that the status of the stocks seems to have been stable or improving depending on the areas. Furthermore, it concluded that, since the reduced MCRS for Venus shells is still larger than the size at maturity (previously reported between 15 and 17 mm), it will probably not be detrimental to the reproductive capacity of the stock and is likely to have little effect on the exploitation rate on juveniles [75]. Therefore, our estimated TL_50_ at an even smaller size supports what stated by STECF.

Moreover, the EU Commission has considered that, based on information available in the Joint Recommendations and STECF assessments [75, 76], the derogation to the MCRS is in line with the objectives of the sustainable exploitation of the Venus shells stock in the Italian territorial waters. The lower MCRS also contributed to reduce the impact of the fishing activity on the marine ecosystem by allowing a significant decrease in fishing time and in the area being dredged as the quota is reached faster. On this basis, it appears that the proposed reduced MCRS would comply with the requirements established for technical measures in Article 15 and Article 18 of Regulation (EC), 2019/1241 [77].

However, there is necessity to:

(i) collect accurate fishery data on fishing effort through the implementation of automatic monitoring system (GPS device) onboard each vessel; the boats’ movement control by the bodies in charge for the inspections (Coast Guard) would allow the coastal area within 0.3 NM of the coast to be preserved in an inexpensive way from illegal fishing activities, and therefore to safeguard a large fraction of the reproductive stocks;
(ii) conduct at least two annual samplings. This would allow to constantly monitor the resource and to relate the biomass landed with those present at sea (exploitation rate). This index, calculated for each Consortium, would be essential to ensure rational and sustainable exploitation. When a threshold value is exceeded, effort management measures and targeted closures should be put in place [6]. Such close monitoring would make it possible to immediately verify any situations of overexploitation.

Populations of marine bivalves are subject to large interannual fluctuations as a result of their sensitivity to unfavourable environmental conditions [78]. Along the Italian Adriatic coasts, extensive dying-off phenomena for *C. gallina* took place several times in the last 30 years [6]. Although it is not always easy to identify the causes of these mass mortality events, they are generally caused by sudden changes in the coastal environment (e.g. hypo-anoxia, fresh water inputs, sea storms, pollution, sudden temperature and seabed grain size variations) and presence of pathogen agents [6]. Considering that the physical and chemical parameters of the seas are changing due to water acidification, global warming, sea level rise and decreased nutrient availability [79–81], the environmental perturbations are likely to frequently raise increasing the pressure on the species. For example, Huntley & Scarponi [82] found an association between sea level rise and increasing prevalence of digenean trematodes in *C. gallina* fossil records from a Holocene shallow marine succession in the Po coastal plain. Moreover, Delgado & Silva [83] noted that, where levels of prevalence of diagenetic-trematode-like parasite were higher they induced castration in the wedge clam (*Donax trunculus*) specimens. However, at present, the possible effects induced by climate change on the life-history traits of *C. gallina* are mainly unknown. For this reason, its main biological traits (e.g. growth, size at sexual maturity, reproductive potential) should be constantly monitored in relation to a changing environment, to guarantee the adoption of suitable management actions for a responsive fishery. Therefore, a careful periodic review of the adopted technical measures based on the biology of the species should be warrant for its protection over time.

Nevertheless, genetic studies [84] confirmed that, despite the fluctuations exhibited by the species in the last four decades, its high level of genetic diversity has not been negatively affected, conferring to this species a good adaptive potential to face the environmental perturbations.

## Conclusions

In conclusion, this study provides some crucial biological information that can help adjust fishery management measures to clam biology. It also confirms that in the Adriatic Sea *C. gallina* reproduces in spring-summer, thus supporting the adoption of fishing closures in this period: closures ensure that the larger individuals contribute to reproduction and that the offspring attach to the substrate. *Chamelea gallina* reaches sexual maturity in the first year of life and partial fecundity is size-related. Even though the MCRS reduction to 22 mm TL affects partial fecundity (specimens measuring 25 mm TL produce 40% more oocytes per female), we suggest that the ability of Adriatic clam stocks to withstand the strong fishing pressure of the past 40 years and the present one is due to their high reproductive potential, multiple spawning events and high genetic variability combined with the effect of management measures (closed areas/seasons, quota, MCRS) and technical constraints on the gear and the sieve on board.

## Methods

### Sample and data collection

**Fig. 8:**
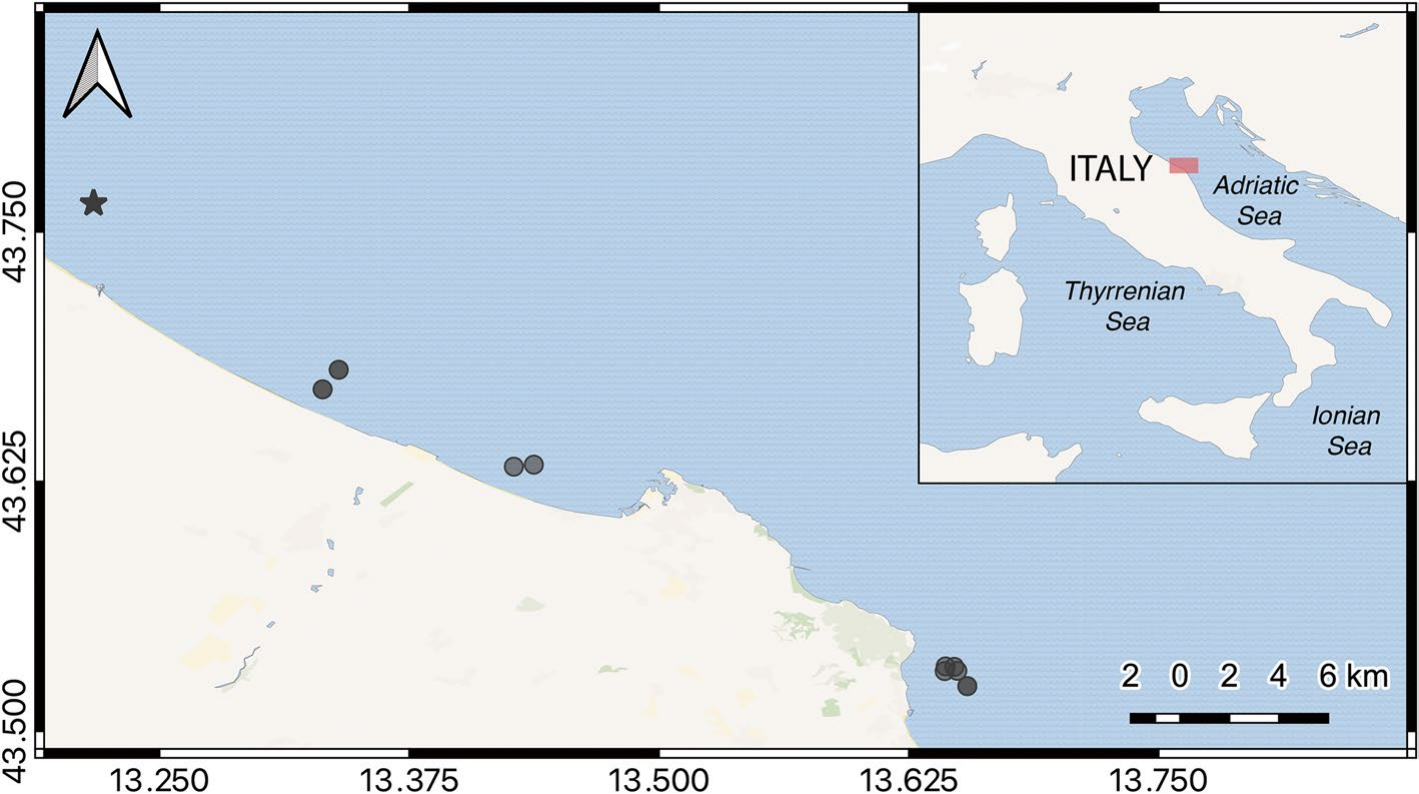
Map of the sampling area generated through the QGIS software version 3.20 “Odense” (www.qgis.org). Dots indicate the sampling positions. The star marks the Tele–Senigallia research pylon, where the bottom seawater temperature data were recorded

Clam samples were collected monthly, from November 2018 to October 2019, during commercial fishing operations conducted on sandy bottoms (depth, 5 to 12 m) in the Ancona Maritime District (central Adriatic Sea, Fig. 8). From 2 to 3 individuals per size class (width, 2 mm) were measured to the nearest 0.1 mm with a Vernier calliper along the anterior-posterior shell axis. The number of specimens each month analysed depended on the size classes available in the sample (maximum size range 18–36 mm TL, overall mean size (± Standard Deviation, SD) 25.4 ± 3.8 mm TL). Testing for differences in the gametogenic cycle in relation to shell size was not considered, however a gonad fragment from each specimen was placed in Dietrich solution [85] for subsequent histological analysis.

Bottom seawater temperature (BST) data were obtained from the Tele–Senigallia pylon, a research tower located 1.3 NM off Senigallia, which is close to the sampling area and has been collecting oceanographic data since 1988 [86]. Temperature data were recorded every 10 min on a daily scale at 12.5 m depth. The daily Chl-a values were freely downloaded from the EU Copernicus Marine Service Information website [87] by tracing a polygon overlapping with the sampling area. Mean monthly values were than calculated for both parameters to observe how in parallel gonadal maturity stages changed over months.

### Histology

The gametogenic cycle of females and males was investigated using a standard histological protocol. Gonads were dehydrated through increasing ethanol concentrations and embedded in paraplast. Serial 6-μm-thick transverse sections were cut with a microtome, mounted on slides, stained with Harris haematoxylin and eosin [88] and finally examined under a light microscope at 5–40 × magnification. Maturity stages were assigned according to the 6-stage scale proposed by Joaquim et al. (2014) for both sexes, with a slight modification of the last stage, as follows: F1/M1, inactive; F2/M2, early active; F3/M3, late active; F4A/M4A, ripe; F4B/M4B, partially spawned; and F5/M5 regressing (rather than “spent” as in the original scale). When multiple stages coexisted in an individual, the predominant stage was assigned.

### Size at sexual maturity

TL_50_ was determined in specimens obtained from additional samples collected on alternate weeks in the central part of the reproductive season (May to August, 8 additional samples). From 3 to 4 individuals per size class (width, 1 mm) were measured and analysed and the total number of individuals per each sample always varied depending on the size classes available in the sample (maximum size range 4–36 mm TL, overall mean size 18.7 ± 9.5 mm TL). An ad hoc dichotomous maturity scale (1, not sexually mature; 2, sexually mature) was applied to classify specimens based on microscopic features. Gonad material was smeared on slides and examined under a light microscope linked to a video analysis system (Las Image Analysis, Leica). Females were classified as immature if only previtellogenic (immature) oocytes (d_max_ ≤ 60 μm) were detected, and as mature when vitellogenic (mature) oocytes (maximum diameter, d_max_ > 60 μm) began to develop. This threshold (≤ or > 60 μm) was set based on what described by Corni et al. [18, 21] in the Adriatic where *C. gallina* previtellogenic oocytes had a different shape (more irregular) and dimension (d_max_ ≤ 60 μm) compared to vitellogenic ones. Males were classified as mature/immature based on the presence/absence of spermatozoa with well elongated branched tails. Whenever not possible to assess the sex of small individuals they were classified as indeterminate and excluded from the calculation of TL_50_. TL_50_ was assessed in both sexes by fitting a logistic model to the proportion of mature specimens per size class:

$$y=\frac1{\left(1+exp^{-\left(a+bcx\right)}\right)}$$

where *y* is the relative frequency of mature individuals; *x* the size of individuals, exp. is the basis of the Neperian logarithms, *a* and *b* are the regression constant, using the R package sizeMat [89].

### Partial fecundity

A total number of 26 females (20 in stage 4A and 6 in stage 4B) ranging from 19 to 33 mm TL, collected during the reproductive season, were examined to investigate the relationship of gonad volume (G_v_) with TL, the number of oocytes contained in gonadal tissue and the percent G_v_ occupied by oocytes. PF was assessed in stage 4A females by histological and video analysis methods, to provide an estimate of the number of gametes released in a single release event in relation to TL. Histological analysis and image post-processing took at least 8 h per individual.

All specimens were measured and opened. All the organs (shell, mantle, siphons, gills) except the visceral mass and the foot were removed before storage in Dietrich solution for subsequent histological analysis. A procedure similar to the one described by Delgado et al. [19] was employed for G_v_ calculation. In brief, the entire visceral mass was cut into sections; a 6-μm-thick section every 100 μm was stained with Harris haematoxylin and eosin and viewed under a stereomicroscope connected to a video analysis system (Leica Application Suite V4.12) using reflected light at low magnification (0.76 ×). The area of the gonad (G_a_) was measured in each section using Image J software, which allowed calculating G_v_. In addition, 6 randomly chosen fields per gonad were digitized under a light microscope at 10 × magnification and used to assess the d_min_ (minimum diameter) and d_max_ of oocytes, which were sectioned through the nucleus. Oocyte volume was then calculated assuming cells to be spheroid (O_v_ = 4/3 × π × d_min_ × d_max_^2^). The total oocyte number of each clam was estimated by standardizing the observations from each field to the entire gonad volume. PF was estimated by summing the number of mature oocytes in each 4A female. The relationship between PF and TL was explored by regression analysis.

### Statistical analysis

The percent G_v_ occupied by all types of oocytes was used to test for statistical differences between maturity stages. The use of percentages allowed to control for differences in G_v_ between individuals. One-way analysis of variance (ANOVA) was applied to establish whether 4A and 4B females showed a significantly different mean percent G_v_ occupation. Two-way ANOVA was applied to investigate possible differences in mean percent G_v_ occupation between maturity stages and between oocyte development stages (mature and immature). Before result interpretation, the data were explored to check the assumption of normality, homoscedasticity and independence. All assumptions were met. After the tests, the Tukey HSD test was performed to explore differences among the levels of significant terms.

Analysis of covariance (ANCOVA) was used to test for statistical differences in oocyte number accounting for differences in TL between individuals. Analogously, a two-way experimental design was used to test for the effect of Maturity stage (2 levels: 4A and 4B) and Oocyte development stage (2 levels: mature and immature) controlling for the covariate, TL. Prior to statistical analysis, the data were explored to check the assumption of normality, homoscedasticity, independence, linearity of regression and homogeneity of slopes. Abundance and length data were log-transformed to meet the assumptions. Finally, a simple main effects test was conducted to explore the interaction between the levels of each term.

All statistical analyses and visualizations were produced in R (v 4.0.3; R Core Team [90]).

## Supplementary Information

**Additional file 1: Suppl. Mat 1.** Summarizes the reproductive traits of *Chamelea gallina* described in the present study and by other authors in different geographical areas.

## Data Availability

The datasets generated during and/or analysed during the current study are available from the corresponding author on reasonable request.

## Abbreviations

MCRS: Minimum Conservation Reference Size
NM: Nautical miles
TL: Total length
TL_50_: Size at sexual maturity
BST: Bottom seawater temperature
Chl-a: Chlorophyll-a
PF: Partial fecundity
SD: Standard deviation
d_max_: Maximum diameter
G_v_: Gonad volume
G_a_: Gonad area
O_v_: Oocyte volume
EC: European Commission
EU: European Union
HSD: Honestly significant difference
CI: Confidence interval
STECF: Scientific, Technical and Economic Committee for Fisheries
Repr. season: Reproductive season
Repr. traits: Reproductive traits
CW: Central-western
NE: North-eastern
N: Northern
C: Central
CS: Central-southern
S: Southern
SW: South-western
NW: North-western
MPS: Multiple partial spawner
Inter-IA: Interindividual asynchrony
Intra-IA: Intraindividual asynchrony
histo: Histological analysis
micro: Microscopic analysis

## References

[1] Morello EB, Froglia C, Atkinson RJA, Moore PG (2006). The effects of hydraulic dredging on the reburial of several molluscan species. Biol Mar Mediterr.

[2] Lucchetti A, Sala A (2012). Impact and performance of Mediterranean fishing gear by side-scan sonar technology. Can J Fish Aquat Sci.

[3] Pérès JM, Picard J (1964). New manual for benthic bionomics in the Mediterranean Sea. Trav la Stn Marittime Endoume.

[4] Romanelli M, Cordisco CA, Giovanardi O (2009). The long-term decline of the *Chamelea gallina* L.(Bivalvia: Veneridae) clam fishery in the Adriatic Sea: is a synthesis possible. Acta Adriat.

[5] Wittmer JM, Dexter TA, Scarponi D, Amorosi A, Kowalewski M (2014). Quantitative bathymetric models for late Quaternary transgressive-regressive cycles of the Po Plain, Italy. J Geol.

[6] DGPEMAC. The National Management Plan for fishing with hydraulic dredges and boat-operated shell-rakes as identified in the classification of fishing equipment use by mechanical dredges including mechanised dredges (HMD) and boat dredges (DRB). Public Law No. 9913 17/06/2019. Ministry for Agricultural, Food and Forestry Policies (MiPAAF). Rome, Italy; 2019.

[7] Carlucci R, Piccinetti C, Scardi M, Del Piero D, Mariani A (2015). Evaluation of the effects on the clam resource in the light of a new minimum landing size and a better biological and commercial management of the product. Final Report: 76.

[8] Lucchetti A, Piccinetti C, Meconi U, Frittelloni C, Marchesan M, Palladino S (2014). Transferable fishing concessions (TFC): a pilot study on the applicability in the Mediterranean Sea. Mar Policy.

[9] Italian Ministry. Ministerial Decree (DM) of 22 December 2000 Subject: Discipline for fishing for bivalve molluscs. Changes to the Ministerial Decree 21.7.98 being registered at the Central Budget Office. 2000:14.

[10] European Council. Council Regulation (EC) No 1967/2006 of 21 December 2006 concerning management measures for the sustainable exploitation of fishery resources in the Mediterranean Sea, amending Regulation (EEC) No 2847/93 and repealing Regulation (EC) No 1626/94. Official Journal of ther European Union, L 409/11. 2006:75.

[11] European Council (2020). Commission Delegated Regulation (EU) 2020/2237 of 13 august 2020 amending Delegated Regulation (EU) 2020/3 as regards the derogation for the Minimum Conservation Reference Size of Venus shells (Venus spp.) in certain italian territorial waters. Off J Eur Union.

[12] European Council. Commission Delegated Regulation (EU) 2016/2376 of 13 October 2016 establishing a rejection plan for bivalve molluscs Venus spp. in Italian territorial waters. Official Journal of ther European Union, L 352/48. 2016:2.

[13] European Council. Commission Delegated Regulation (EU) 2020/3 of 28 August 2019 establishing a discard plan for Venus shells (Venus spp.) in certain Italian territorial waters. Official Journal of the European Union, L2/1. 2020:4.

[14] Valli G, Zecchini-Pinesich G (1981). Considerations on biometrics and the reproduction of *Chamelea gallina* (L) (Mollusca, Bivalvia) from the Gulf of Trieste (Upper Adriatic). Nov Thalass.

[15] Cordisco CA, Romanelli M, Trotta P. Annual distribution and description of the larval stages of *Chamelea gallina* and *Mytilus galloprovincialis* in the central-southern Adriatic. Assoc Ital di Oceanol e Limnol. 2003;16:93–103.

[16] Scopa M, Nerone E, Recchi S, Barile NB (2014). Trends in the *Chamelea gallina* Production from Molise Region (Adriatic Sea, Italy): A Ten-Year Survey. Glob J Sci Front Res Agric Vet.

[17] Poggiani L, Piccinetti C, Piccinetti MG (1973). Observations on the biology of bivalve molluscs *Venus gallina* L. and *Tapes aureus* Gmelin in the northern Adriatic. Note del Lab di Biol Mar e Pesca. Fano..

[18] Corni MG, Cattani O, Mancini L, Sansoni G. Aspects of the *Venus gallina* L. life cycle in relation to the protection of existing stocks. (In Italian). Pubbl a cura del Consorzio per Cent Univ di Stud e Ric sulle Risorse Biol Mar di Cesenatico. 1980:2–12.

[19] Delgado M, Silva L, Juárez A (2013). Aspects of reproduction of striped venus *Chamelea gallina* in the Gulf of Cádiz (SW Spain): implications for fishery management. Fish Res.

[20] Marano G, Casavola N, Saracino C, Rizzi E (1982). Reproduction and growth of *Chamelea gallina* (L.) and *Venus verrucosa* (L.) (Bivalvia: Veneridae) in the southern Adriatic. Mem Biol Mar Oceanogr.

[21] Corni MG, Farneti M, Scarselli E (1985). Histomorphological aspects of the gonads of *Chamelea gallina* (Linné) (Bivalvia: Veneridae) in autumn. J Shellfish Res.

[22] Dalgiç G, Karayucel S, Okumua I (2009). Reproduction cycle of striped venus *Chamelea gallina* from the Black Sea coast of Turkey. J Anim Vet Adv.

[23] Rodríguez De La Rúa A, Prado MA, Bruzón MA (2011). Study of the reproductive cycle of *Chamelea gallina* (L., 1758) (Mollusca: Bivalvia) in three populations of the Andalusian coast. Bol Inst Esp Oceanogr.

[24] Joaquim S, Matias D, Matias AM, Moura P, Roque C, Chícharo L (2014). Biochemical and energy dynamics throughout the reproductive cycle of the striped venus *Chamelea gallina* (Mollusca, Bivalvia). Invertebr Reprod Dev.

[25] Chavez-Villalba J, Barret J, Mingant C, Cochard J-C, Le Pennec M (2003). Influence of timing of broodstock collection on conditioning, oocyte production, and larval rearing of the oyster, *Crassostrea gigas* (Thunberg), at six production sites in France. J Shellfish Res.

[26] Braley RD (1982). Reproductive periodicity in the indigenous oyster Saccostrea cucullata in Sasa Bay, Apra Harbor. Guam Mar Biol.

[27] Kautsky N (1982). Quantitative studies on gonad cycle, fecundity, reproductive output and recruitment in a Baltic *Mytilus edulis* population. Mar Biol.

[28] Thompson RJ, Newell RIE, Kennedy VS, Mann R. Reproductive processes and early development. East oyster *Crassostrea virginica*. 1996:335–370.

[29] Llodra ER. Fecundity and life-history strategies in marine invertebrates. Adv Mar Biol. 2002;43:87–170. 10.1016/s0065-2881(02)43004-0 12154615

[30] Hamel J-F, Conand C, Pawson DL, Mercier A (2001). The sea cucumber *Holothuria scabra* (Holothuroidea: Echinodermata): Its biology and exploitation as beche-de-mer. Adv Mar Biol.

[31] Utting SD, Millican PF (1997). Techniques for the hatchery conditioning of bivalve broodstocks and the subsequent effect on egg quality and larval viability. Aquaculture..

[32] Fong PP, Deguchi R, Kyozuka K (1996). Serotonergic ligands induce spawning but not oocyte maturation in the bivalve Mactra chinensis from central Japan. Biol Bull.

[33] Pouvreau S, Gangnery A, Tiapari J, Lagarde F, Garnier M, Bodoy A (2000). Gametogenic cycle and reproductive effort of the tropical blacklip pearl oyster, *Pinctada margaritifera* (Bivalvia: Pteriidae), cultivated in Takapoto atoll (French Polynesia). Aquat Living Resour.

[34] Hendriks IE, Van Duren LA, Herman PMJ (2005). Image analysis techniques: A tool for the identification of bivalve larvae?. J Sea Res.

[35] Hendriks IE, van Duren LA, Herman PMJ (2003). Effect of dietary polyunsaturated fatty acids on reproductive output and larval growth of bivalves. J Exp Mar Biol Ecol.

[36] Galinou-Mitsoudi S, Sinis AI (1994). Reproductive cycle and fecundity of the date mussel Lithophaga lithophaga (Bivalvia: Mytilidae). J Molluscan Stud.

[37] Moles KR, Layzer JB (2008). Reproductive ecology of *Actinonaias ligamentina* (Bivalvia: Unionidae) in a regulated river. J North Am Benthol Soc.

[38] Mzighani S (2005). Fecundity and population structure of cockles, Anadara antiquata L. 1758 (Bivalvia: Arcidae) from a sandy/muddy beach near Dar es Salaam, Tanzania. West Indian Ocean J Mar Sci.

[39] Joaquim S, Matias D, Matias AM, Moura P, Arnold WS, Chícharo L (2011). Reproductive activity and biochemical composition of the pullet carpet shell *Venerupis senegalensis* (Gmelin, 1791)(Mollusca: Bivalvia) from Ria de Aveiro (northwestern coast of Portugal). Sci.

[40] Kang S-G, Choi K-S, Bulgakov AA, Kim Y, Kim S-Y (2003). Enzyme-linked immunosorbent assay (ELISA) used in quantification of reproductive output in the pacific oyster, *Crassostrea gigas*, in Korea. J Exp Mar Biol Ecol.

[41] Park K-I, Choi K-S (2004). Application of enzyme-linked immunosorbent assay for studying of reproduction in the Manila clam Ruditapes philippinarum (Mollusca: Bivalvia): I. Quantifying eggs. Aquaculture..

[42] Erkan M (2009). Ultrastructure of ovary and oogenesis in *Chamelea gallina* (Linné, 1758) (Bivalvia, Veneridae). Invertebr Reprod Dev.

[43] Plough LV, Shin G, Hedgecock D (2016). Genetic inviability is a major driver of type III survivorship in experimental families of a highly fecund marine bivalve. Mol Ecol.

[44] Johnson KD, Smee DL (2012). Size matters for risk assessment and resource allocation in bivalves. Mar Ecol Prog Ser.

[45] Honkoop PJC, Van der Meer J, Beukema JJ, Kwast D (1998). Does temperature-influenced egg production predict the recruitment in the bivalve *Macoma balthica*?. Mar Ecol Prog Ser.

[46] Cordisco CA, Trotta PMR (2005). Reproductive plasticity of the common clam *Chamelea gallina* (Linnaeus, 1758). Biol Mar Mediterr.

[47] Rizzo G, Cernigai F, Marceta T, Bressan M, Marin MG (2011). Physiological and reproductive features in *Chamelea gallina* as a contribution to stock management in the northern Adriatic Sea. Biol Mar Mediterr.

[48] Dang C, De Montaudouin X, Gam M, Paroissin C, Bru N, Caill-Milly N (2010). The Manila clam population in Arcachon Bay (SW France): can it be kept sustainable?. J Sea Res.

[49] Froglia C (1975). Observations on the growth of *Chamelea gallina* (L.) and *Ensis minor* (Chenu) in the middle Adriatic. Quad di Lab di Tecnol della Pesca.

[50] Salvatorelli G (1967). Observations on the annual reproductive cycle of *Venus gallina* (Lamellibranch Molluscs). Ann dell’Università di Ferrara (Nuova Ser Sez XIII). Anat Comp.

[51] Darriba S, San Juan F, Guerra A (2004). Reproductive cycle of the razor clam *Ensis arcuatus* (Jeffreys, 1865) in northwest Spain and its relation to environmental conditions. J Exp Mar Biol Ecol.

[52] Dridi S, Romdhane MS, Elcafsi M (2007). Seasonal variation in weight and biochemical composition of the Pacific oyster, *Crassostrea gigas* in relation to the gametogenic cycle and environmental conditions of the Bizert lagoon, Tunisia. Aquaculture..

[53] Enríquez-Díaz M, Pouvreau S, Chávez-Villalba J, Le Pennec M (2009). Gametogenesis, reproductive investment, and spawning behavior of the Pacific giant oyster *Crassostrea gigas*: evidence of an environment-dependent strategy. Aquac Int.

[54] Ojea J, Pazos AJ, Martınez D, Novoa S, Sanchez JL, Abad M (2004). Seasonal variation in weight and biochemical composition of the tissues of Ruditapes decussatus in relation to the gametogenic cycle. Aquaculture..

[55] Yan H, Li Q, Yu R, Kong L (2010). Seasonal variations in biochemical composition and reproductive activity of Venus Clam Cyclina sinensis (Gmelin) from the yellow River Delta in Northern China in Relation to Environmental Factors. J Shellfish Res.

[56] Yan H, Li Q, Liu W, Yu R, Kong L (2010). Seasonal changes in reproductive activity and biochemical composition of the razor clam Sinonovacula constricta (Lamarck 1818). Mar Biol Res.

[57] Da Costa F, Aranda-Burgos JA, Cerviño-Otero A, Fernandez-Pardo A, Louzán A, Nóvoa S, et al. Clam reproduction. In: Clam Fisheries and Aquaculture. New York: Nova Science Publisher; 2013. p. 45–71.

[58] Normand J, Le Pennec M, Boudry P (2008). Comparative histological study of gametogenesis in diploid and triploid Pacific oysters (*Crassostrea gigas*) reared in an estuarine farming site in France during the 2003 heatwave. Aquaculture..

[59] Bratoš Cetinić A, Gavrilović A, Dupčić Radić I, Pećarević M, Tomšić S, Marčelja E, et al. Reproductive characteristics of baby clam Chamelea gallina Linnaeus, 1758 (Bivalvia, Mollusca) from the river Neretva estuary. Proceedings 42nd Croatian and 2nd International symposium on agriculture: Opatija (Croatia), February 13-16, 2007. In: Zbornik sažetaka. Opatija, Croatia; 2007. p. 192–3.

[60] Gaspar MB, Monteiro CC (1998). Reproductive Cycles of the razor clam *Ensis Siliqua* and the clam *Venus Striatula* off Vilamoura, Southern Portugal. J Mar Biol Assoc United Kingdom.

[61] Bargione G, Vasapollo C, Donato F, Virgili M, Petetta A, Lucchetti A (2020). Age and Growth of Striped Venus Clam *Chamelea gallina* (Linnaeus, 1758) in the Mid-Western Adriatic Sea: A Comparison of Three Laboratory Techniques. Front Mar Sci.

[62] Giansante C, Angelini L, Angioni SA, Biase P, Di Giacomandrea A, Gatti G, et al. Management and protection of the natural banks of *Chamelea gallina. *Abruzzo Region: (Adriatic clam) in the Pescara Maritime Department; 2006. p. 181. https://www.izs.it/IZS/Engine/RAServeFile.php/f/Docup_Pesca_Relazioni_Scientifiche/Relazioni_Scientifiche_4.6/IZS_sfop_vongole.pdf

[63] Delgado M, Pérez CA (2003). A study of gonadal development in Ruditapes decussatus (L.)(Mollusca, Bivalvia), using image analysis techniques: influence of food ration and energy balance. J Shellfish Res.

[64] Silva L, Juárez A (2009). Study on the striped venus clam (*Chamelea gallina*) fishing with hydraulic dredgers and towed rakes in the fishing ground of the Gulf of Cádiz.

[65] Beninger PG, Chérel D, Kessler L. Examining bivalve fecundity: oocyte viability revealed by Neutral Red vital staining. Aquac Int. 2021;29:1219–31.

[66] Murua H, Kraus G, Saborido-Rey F, Witthames P, Thorsen A, Junquera S (2003). Procedures to estimate fecundity of marine fish species from field samples in relation to reproductive strategy. J Northwest Atl Fish Sci.

[67] Morvan C, Ansell AD (1988). Stereological methods applied to reproductive cycle of *Tapes rhomboides*. Mar Biol.

[68] Soria G, Tordecillas-Guillen J, Cudney-Bueno R, Shaw W (2010). Spawning induction, fecundity estimation, and larval culture of Spondylus calcifer (Carpenter, 1857)(Bivalvia: Spondylidae). J Shellfish Res.

[69] Krug PJ (1998). Poecilogony in an estuarine opisthobranch: planktotrophy, lecithotrophy, and mixed clutches in a population of the ascoglossan *Alderia modesta*. Mar Biol.

[70] André C, Lindegarth M (1995). Fertilization efficiency and gamete viability of a sessile, free-spawning bivalve, *Cerastoderma edule*. Ophelia..

[71] Cushing DH (1990). Plankton production and year-class strength in fish populations: an update of the match/mismatch hypothesis. Advances in marine biology.

[72] Cowen RK, Paris CB, Srinivasan A (2006). Scaling of connectivity in marine populations. Science (80-).

[73] Sala A, Brčić J, Herrmann B, Lucchetti A, Virgili M. Assessment of size selectivity in hydraulic clam dredge fisheries. Can J Fish Aquat Sci. 2017;74.

[74] Bargione G, Petetta A, Vasapollo C, Virgili M, Lucchetti A (2021). Reburial potential and survivability of the striped venus clam (*Chamelea gallina*) in hydraulic dredge fisheries. Sci Rep.

[75] STECF (2020). Scientific, Technical and Economic Committee for Fisheries – 63rd Plenary Report – Written Procedure (Plen-20-01).

[76] STECF (2019). Scientific, Technical and Economic Committee for Fisheries (STECF) - Multiannual Plan for the fisheries exploiting demersal stocks in the Adriatic Sea (STECF-19-02).

[77] European Council. Regulation (EU) 2019/1241 of the European Parliament and of the Council of 20 June 2019 on the conservation of fisheries resources and the protection of marine ecosystems through technical measures, amending Council Regulations (EC) No 1967/2006, (EC) No. 2019:97.

[78] Rufino MM, Vasconcelos P, Pereira F, Moura P, Gaspar MB (2018). Bivalve sanctuaries to enhance stocks along the Algarve coast of southern Portugal: A spatio-temporal approach. Aquat Conserv Mar Freshwat Ecosyst.

[79] Doney SC (2006). The dangers of ocean acidification. Sci Am.

[80] Belkin IM (2009). Rapid warming of large marine ecosystems. Prog Oceanogr.

[81] Wohlers J, Engel A, Zöllner E, Breithaupt P, Jürgens K, Hoppe H-G (2009). Changes in biogenic carbon flow in response to sea surface warming. Proc Natl Acad Sci.

[82] Huntley JW, Scarponi D (2021). Parasitism and host behavior in the context of a changing environment: The Holocene record of the commercially important bivalve *Chamelea gallina*, northern Italy. PLoS One.

[83] Delgado M, Silva L (2018). Timing variations and effects of size on the reproductive output of the wedge clam Donax trunculus (L. 1758) in the littoral of Huelva (SW Spain). J Mar Biol Assoc United Kingdom.

[84] Carducci F, Biscotti MA, Trucchi E, Giuliani ME, Gorbi S, Coluccelli A (2020). Omics approaches for conservation biology research on the bivalve *Chamelea gallina*. Sci Rep.

[85] Gray P (1954). The microtomist’s formulary and guide.

[86] Ravaioli M, Bergami C, Riminucci F, Langone L, Cardin V, Di Sarra A (2016). The RITMARE Italian Fixed-Point Observatory Network (IFON) for marine environmental monitoring: A case study. J Oper Oceanogr.

[87] E.U. Copernicus Marine Service Information. http://marine.copernicus.eu. Accessed 1 Jun 2021.

[88] Pearse AGE. Analytical Tehnology. Histochem Theor Appl vol 2 Churchill Livingstone. 1985;2:726.

[89] Torrejon-Magallanes J (2019). sizeMat: an R package to estimate size at sexual maturity. CRAN R-Project.

[90] R Core Team (2020). R: A language and environment for statistical computing.

