# Supplementary material for: Chamelea gallina reproductive biology and Minimum Conservation Reference Size: implications for fishery management in the Adriatic Sea
Source: BMC Zool. 2021 Nov 25;6:32. doi: 10.1186/s40850-021-00096-4 (PMC10124184; doi:10.1186/s40850-021-00096-4)
Supplement: Supplementary file 1 — Additional file 1: Suppl. Mat 1. Summarizes the reproductive traits of Chamelea gallina described in the present study and by other authors in different geographical areas. [file 40850_2021_96_MOESM1_ESM.docx]

***Chamelea gallina* reproductive biology and Minimum Conservation Reference Size: implications for fishery management in the Adriatic Sea**

**Journal name: BMC Zoology**

Giada Bargione^1,2^*, Fortunata Donato^1^, Giulio Barone^1^, Massimo Virgili^1^, Pierluigi Penna^1^, Alessandro Lucchetti^1^

1. *National Research Council (CNR), Institute for Biological Resources and Marine Biotechnologies (IRBIM); Largo Fiera della Pesca, 1, 60125 Ancona, Italy*
2. *Department of Biological, Geological and Environmental Sciences, University of Bologna, Piazza di Porta San Donato 1, 40126 Bologna, Italy*

* Corresponding author.

*E-mail address*: giada.bargione@irbim.cnr.it

Additional file 1. Reproductive traits of Chamelea gallina described in the present study and by other authors in different geographical areas. (Repr. season = Reproductive season, Repr. traits = Reproductive traits, CW = central-western, NE = north-eastern, N = northern; C = central, CS = central-southern, S = southern, SW = south-western, NW = north-western, MPS = multiple partial spawner, Inter-IA = interindividual asynchrony, Intra-IA = intraindividual asynchrony, histo = histological analysis, micro = microscopic analysis, blank cells = info not available).

| **Reference** | **Studied Area** | **Period investigated** | **Onset gametogenesis** | **Spawning period** | **Rep. Seas. Duration** | **TL50** | **Rep. Traits** | **Methods** |
| --- | --- | --- | --- | --- | --- | --- | --- | --- |
| **Present study** | **CW Adriatic (Ancona)** | **Nov 2018-Oct2019** | **Nov** | **May-Sept** | **prolonged** | **11.2 (F), 11.8 (M)** | **MPS, Inter-IA, Intra-IA** | **histo + micro** |
| Bratoš Cetiniæ et al. 2007 | NE Adriatic (Croatia, river Neretva) | Jan 2003 - Jan 2004 | Oct-Nov | Jan-Aug | prolonged | 12 |  | histo |
| Salvatorelli 1967 | N Adriatic (Chioggia Lagoon) | 1967 | Oct (F), Dec (M) | Jul-Aug | limited |  | Inter-IA | histo |
| Corni et al. 1985a | N Adriatic (Cesenatico) | May-Jun 1980 |  | Jun | prolonged | 10 (F), 11 (M) | MPS, Inter-IA | histo + micro |
| Corni et al. 1985b | N Adriatic (Cesenatico) | Sept-Dec 1982 | Oct (F), Oct-Dec (M) | Sept-Oct |  |  | Inter-IA, Intra-IA | histo |
| Poggiani et al. 1973 | N Adriatic | Jul 1968 - Mar 1970 | Nov | Jun-Jul | limited | 16-18 | Inter-IA | histo |
| Valli & Zecchini-Pinesich 1981 | N Adriatic (Trieste) | Apr 1975-Apr 1976 | Oct | Apr-Sept | prolonged |  | MPS, Inter-IA | histo |
| Valli et al. 1985 | N Adriatic (Grado) | 1981-1982 | Sept | Apr-Jul | limited |  |  | histo |
| Ambrogi et al. 1997 | N Adriatic (Po' delta) | 1997 |  | May-Nov | prolonged |  |  |  |
| Rizzo et al 2011 | N Adriatic (Chioggia Lagoon ) | Jun 2009 - May 2010 | Sept-Oct | Jun-Aug | limited |  |  | micro |
| Moschino & Marin 2006 | N Adriatic (Chioggia and Venezia) | 2000-2001 |  | spring - summer |  |  |  | CI |
| Franceschini e Bernarello 2013 | N Adriatic (Porto Viro) | 2013 | Apr-Oct | Apr-Oct | prolonged |  |  |  |
| Froglia 1975 | C Adriatic |  |  | May-Jul, Sept-Oct | limited |  |  | micro |
| Giansante et al. 2006 | C Adriatic (Pescara) | 2005-2006 |  | summer | prolonged | 12 |  |  |
| Cordisco et al. 2003 | CS Adriatic | 2000-2002 |  | late spring - summer | |  | Inter-IA |  |
| Cordisco et al. 2005 | CS Adriatic (Molise) | 2000-2001 | late-winter/early spring | late spring +autumn | prolonged | 13 | MPS | micro |
| Romanelli 2009 | Adriatic Sea |  |  | Apr-Oct | prolonged | 13-15 |  |  |
| Scopa et. al 2014 | CS Adriatic (Abruzzo, Molise) |  |  | spring-summer |  | 13-15 |  |  |
| Marano et al. 1982 | S Adriatic (Gulf of Manfredonia) | 1978-1979 | Aug-Sept | Jun-Aug | prolonged |  | MPS, Intra-IA | histo + micro |
| Cordisco et al. 2005 | C Tyrrhenian Sea (Lazio) | 2003 | - | Aprl-Nov | prolonged |  | MPS, Inter-IA | micro |
| Gaspar & Monteiro 1998 | S Portugal | Jun 1992 - May 1993 | Oct - Nov | Apr-Aug |  |  | Inter-IA | histo |
| Joaquim et al. 2014 | S Portugal (Algarve coast) | 2009 | Dec | May-Sept | prolonged |  | Inter-IA | histo |
| Vizuete et al. 1993 | S Spain (Mazarrón Bay) | Sept 1987 - Nov 1988 | Dec | May-Sept | prolonged |  | Inter-IA | micro |
| Delgado et al. 2013 | SW Spain (Gulf of Cádiz) | May 2010 - Apr 2011 | Nov-Feb | May-Aug | prolonged | 10.29 (F); 8.41 (M); 9.34 (TOT) | MPS, Inter-IA, Intra-IA | histo |
| Silva & Juàrez 2009 | SW Spain (Gulf of Cádiz) |  |  |  |  | 9 |  |  |
| Rodriguez de la Rùa et al. 2003 | S Spain (Atlantic) | Jun 1999 - May 2000 | Nov | Jan-Sept | prolonged |  | Inter-IA | histo |
| Rodriguez de la Rùa et al. 2003 | S Spain (Mediterranean) | Jun 1999 - May 2000 |  | almost all year | prolonged |  | Inter-IA |  |
| Ramon Herrero 1990 | NW Mediterranean (Valencia, Spain) | May 1988 - May 1990 | Dec | Jun-Aug | limeted |  | Inter-IA | micro |
| Dalgic et al. 2009 | Black Sea | Dec 2002 - Nov 2003 | Mar-May | Jun-Aug | limited |  | Inter-IA | histo |
| Oray et al. 1991 | Marmara Sea |  |  | Jun-Jul |  |  |  |  |
| Erkan 2002 | Marmara Sea |  |  |  |  | 18 | MPS |  |
|  |  |  |  |  |  |  |  |  |
